# Hybrid Nanogels: Stealth and Biocompatible Structures for Drug Delivery Applications

**DOI:** 10.3390/pharmaceutics11020071

**Published:** 2019-02-07

**Authors:** Parisa Eslami, Filippo Rossi, Stefano Fedeli

**Affiliations:** 1 Laboratory of Molecular Magnetism (LaMM), Department of Chemistry “Ugo Shiff”, University of Florence, via della Lastruccia 3, 50019 Sesto Fiorentino, Italy; parisa.eslami@unifi.it; 2 Department of Chemistry, Materials and Chemical Engineering “Giulio Natta”, Politecnico di Milano, via Mancinelli 7, 20131 Milano, Italy; filippo.rossi@polimi.it; 3 Colorobbia Research Center (CERICOL), via Pietramarina 53, 50053 Sovigliana Vinci, Italy

**Keywords:** nanogels, nanohybrids, stealth nanoparticles, biocompatible carriers, drug delivery, PEGylation, zwitterionic polymers, nanolipogels

## Abstract

Considering nanogels, we have focused our attention on hybrid nanosystems for drug delivery and biomedical purposes. The distinctive strength of these structures is the capability to join the properties of nanosystems with the polymeric structures, where versatility is strongly demanded for biomedical applications. Alongside with the therapeutic effect, a non-secondary requirement of the nanosystem is indeed its biocompatibility. The importance to fulfill this aim is not only driven by the priority to reduce, as much as possible, the inflammatory or the immune response of the organism, but also by the need to improve circulation lifetime, biodistribution, and bioavailability of the carried drugs. In this framework, we have therefore gathered the hybrid nanogels specifically designed to increase their biocompatibility, evade the recognition by the immune system, and overcome the self-defense mechanisms present in the bloodstream of the host organism. The works have been essentially organized according to the hybrid morphologies and to the strategies adopted to fulfill these aims: Nanogels combined with nanoparticles or with liposomes, and involving polyethylene glycol chains or zwitterionic polymers.

## 1. Introduction

Among the different polymeric biomaterials studied for application in nanomedicine, a considerable interest is focused on nanogels [1,2,3,4]. These constructs can be described as soft polymeric nanoparticles designed to be stable in a liquid media, typically aqueous, as a dispersed phase. The polymer of the particles is crosslinked, and in the presence of a solvent, these swell turn into stable “jelly” nanoparticles [5]. Such nanostructures are extensively exploited to prepare drug delivery system [6,7] due to their compatibility with the physiological environment [8,9], capability to host drug molecules in their polymeric network [10], and of course, for the adaptability of the involved polymers [11]. The particles can be easily decorated, modified, and functionalized to prepare a very wide variety of architectures [12]. Moreover, due to their versatility, a significant portion of studies concern nanohybrids obtained through their conjugation with other types of nanoparticle and nanostructures, both organic [13] and inorganic [14]. This approach has been studied for many years for nanomedicine application [15], since it allows for the exploitation of the properties derived from the nanoparticles maintaining the characteristics of the nanogel. Several types of particles are involved in hybrid nanogels: Gold nanoparticle [16], carbonaceous materials [17], liposomes [18], quantum dots [19], and magnetic nanoparticles [20,21,22] are just the most common examples.

**Figure 1:**
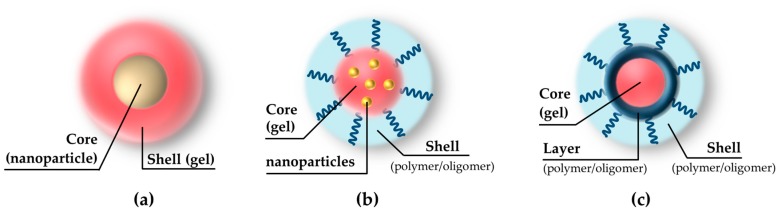
Common hybrid nanogels morphologies: (**a**) Nanoparticle as the core, encapsulated by the nanogel shell; (**b**) core-shell with nanoparticle homogeneously located in the nanogel core and surrounded by a polymeric shell; and (**c**) polymer network assembled in core-layer-shell structure. (Artwork not drawn to scale, the sizes for nanoparticles or nanogels range from 50 to 400 nm).

The morphologies of hybrid nanogels may be multiple, according to the type of involved particles and to the assembly technique [23,24]. Frequently, the nanoparticles represent the core of the hybrid, surrounded by a polymer shell [25,26,27] or homogeneously embedded in the polymer network [28,29]; in other cases, instead, the polymeric structures are themselves assembled in core-layer-shell geometries [30]. The sizes may vary according to the nanohybrid type and to the dimensions of the involved particles, however, as described below, the typical overall range is between 50 and about 400 nm. These morphologies are qualitatively represented in Figure 1. It should be noted, however, how even further combinations are appreciable in the literature [31,32].

To prepare such nanohybrids, many types of reactions may be adopted [33], typically carried out in the dispersed phase [34,35]. However, the radical polymerizations, free or controlled, probably remain the most pursued approach [36,37,38,39]. This class of reactions typically exploits the reactivity of acrylates or vinyl groups, allowing an easy variation of the monomers and crosslinker without substantially redesigning the reaction conditions. In addition, the possibility to choose, among many different types of monomers, favors the selection of the most appropriate functional group for the desired properties [26,40,41]. The polymer network can also be obtained through physical crosslinking. In this case, although the preparation and stability of physical non-covalent nanogels may be more difficult to control or predict, there are still valid examples based on hydrogen bond [42,43,44], ionic interactions [45,46,47], and other intermolecular bonds [48,49,50]. In summary, the overall purpose of hybrid nanogels is to protect the loaded drug and perform a tailored delivery by tuning the interactions with the host environment [51,52], and through a fine design of the structure [53].

Nanostructured drug delivery systems are mostly devised for the cancer treatment through parenteral administration, as the intravenous or the intradermal route [54,55]. Other types of administration like oral, topical, inhalation, and so on, indeed also offers physical barriers to overcome prior to reaching the target; nevertheless, even in trough systemic or local injection, hurdles are still multiple. When a nanoparticle enters a living organism, one of the first factors to consider should be the interaction between the components of blood stream, especially those belonging to immune system, and the nanoparticle’s surface [56,57]. In this regard, several authors draw the attention on the biocorona, an ensemble of proteins and biomolecules that entirely wrap the nanoparticles as these enter the organism, deemed to be the true entity involved in physiological interactions [58]. The result of bio/protein corona formation is a new nano-object which biological fate might be influenced only marginally by the original nanoparticle’s features [59]. In this background, a proper surface decoration, together with a control over dimensions, seems to be a parameter capable of strongly influencing the biodistribution [60,61,62,63]. In any case, the issues are various: Sequestration from the circulation operated by the immune system (opsonization), early clearance, poor pharmacokinetic, and eventually the accumulation far from the desired site of action [64,65,66]. Moreover, a lack of biocompatible surface could induce the host organism to develop inflammatory responses to the introduced substance [67]. As a further concern, although the considered decoration may be mainly aimed to improve the biodistribution/biocompatibility of the particle, even the matter related to the ligands interaction with cancer tissues should be taken into account [68]. Then, considering the role of the dimensions, nanoparticles smaller than 10–20 nm appear to be quickly removed by renal clearance, while those larger than 120–150 nm would be captured by the liver and spleen [69,70]. Even surface charge (zeta) has been identified among the factors affecting the circulating time and the in vivo behavior [71]. Despite various opinions, data suggest that a small negative charge or a neutral surface could be the best conditions to prolong the circulating time of nanostructures [64,72,73].

Concerning hybrid nanogels, we reviewed here the different nanohybrids equipped with functionalizations or strategies tailored to tackle the opsonization and the unfavorable conditions of a living system. For the drug delivery, we actually consider a critical challenge to the development of stealth systems, namely, carriers endowed of the broadest biocompatibility towards the organism or even invisibles to its immune system. The considered hybrid nanogels, whose main morphologies are schematized in Table 1, have therefore been grouped according to the strategy adopted, examining in vitro and in vivo results, approaches, and structures devised to fulfill the aim of biological compatibility. To the state of the art, many stealth functionalizations exploit hydrophilic polymeric chains as polyethylene glycols or chitosan. Nevertheless, in addition to the preparation and decoration methods, the limits of this approach will also be briefly discussed. Even more recent examples, such as zwitterionic polymers and liposome-embedded structures, seem to provide satisfactory stealth properties and have been properly considered. 

## 2. Polyethylene Glycol Decoration 

The surface decoration with polyethylene glycol (PEG) chains, termed PEGylation, was one of the earliest methods employed to provide biocompatibility to nanomaterials entering the organism [86]. This is also the most adopted one on clinically approved drugs, and could be considered as a benchmark of stealth decoration [87]. On nanogels, PEGylated systems have been adopted for many years with appreciable results on drug delivery application [88]. The successful mechanisms of PEGylation may be due to multiple factors: PEG hydrophilic chains, typically with M_w_ between 2.000 and 13.000 Da [89], create a hindered zone around the nanoparticle that reduce the wrapping by plasma proteins and the subsequent uptake by macrophages [90]. Another main effect is related to the high hydrophilicity acquired by the particle surface, less susceptible to antibody recognition [91]. This effect is even described as the formation of an aqueous layer that disguise the structure from the opsonization processes [92]. According to the ligand density and polymer sizes, the chains may assume different spatial dispositions (e.g., “mushroom” or “brush”). Nevertheless, a high density [93] and lengths of at least of 5 kDa seem to provide better outcomes [94]; overall, the observed effect of PEGylation is to increase the bioavailability of decorated nanostructures by extending the circulating lifetime [95]. Thanks to the numerous studies, it was also possible to identify other effects arising from this decoration; authors observed variations in the drug release rate [96] or even an improvement of the uptake by cancer cells due to a specific protein corona, formed around the antifouling PEG functionalization [73]. Other works suggest the adoption of cleavable chains, detachable after endocytosis, to speed up the endosomal escape [90]. Such an effect is also to remove it from the particles possible obstacles to the cellular internalization, once these are extravasated from blood vessels [97]. Another discovered effect, actually related to the extended circulating lifetime, is the rise of an immune response PEG-specific. This was observed with repeated administration of the compound, which resulted in liver and spleen accumulation, in addition to a rapid clearance of the material [98].

**Figure 2:**
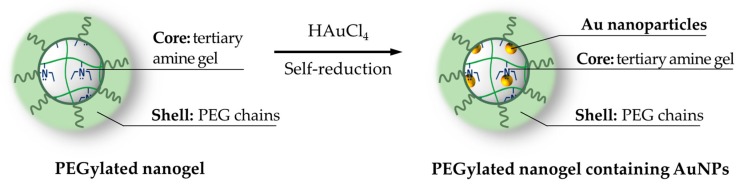
Structure of hybrid PEGylated nanogel prepared by in situ self-reduction of HAuCl_4._

A pioneer effort on hybrid PEGylated nanogel should be credited to Nagasaki and coworkers, who have been developing, for several years, hybrid nanogels incorporating gold nanoparticles in the polymer. In an early work [99], they prepared acrylate pH-sensitive nanogels introducing the PEG decoration (M_w_ = 8000 Da) through the comonomer PEG-styrene in the radical emulsion polymerization. While the compound is yet to contain gold and cannot be considered hybrid, it is worth mentioning that biological tests showed absence of toxicity on MCF-7 and HuH-7 cells. Afterwards, they developed a hybrid gold nanoparticle-gel through permeation of HAuCl_4_ in the polymer core and successive self-reduction of the metal, triggered by the amino groups [100] (Figure 2); in this case, the PEGylation was achieved adding PEG-methacrylate as the initial comonomer. The nanohybrid shows a diameter near 70 nm at neutral pH, evaluated by Dynamic Light Scattering (DLS). 

**Figure 3:**
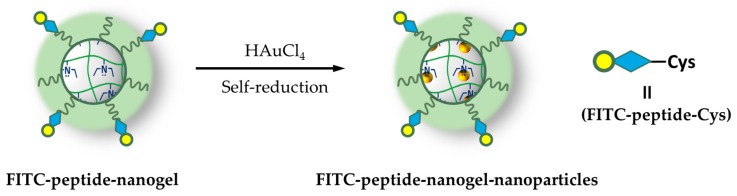
The PEGylated nanogel containing gold nanoparticles and fluorescein isothiocyanate-labeled peptides.

A noteworthy variation, schematized in Figure 3, has been obtained by inserting a caspase-3 cleavable and fluorescein isothiocyanate (FITC)-labeled peptide chain [74]. 

In solution, due to the presence of gold nanoparticles, the nanohybrid shows the quenching of the FITC fluorescence. Upon internalization into cancer cells, instead, the presence of caspase-3 cleaves the peptide releasing the FITC and restoring the fluorescence. In vitro results have been carried out on HuH-7 hepatocarcinoma cell line, evidencing the role of PEGylation in providing a gel without the typical biocompatibility issues of polyamine. To extend the work, the research group prepared another nanogel for the encapsulation of gold nanoparticles as a photothermal device against HeLa carcinoma cells [75]. In the absence of laser irradiation, no toxicity was observed for the PEGylated nanogel, even at concentrations up to 480 µg/mL. However, authors show that the toxicity rises up as the crosslinking density decreases, the toxicity rises up, ascribing this result to the exposure of the polyamine branches. This effect is also reported in a work on PEGylated polyamine acrylic nanogel [101], in which the reduced biocompatibility is attributed to an insufficient length of PEG chains (1500 instead of 8000 Da). In the successive years, although without biological tests, Nagasaki et al. also successfully incorporated silica/gold nanoparticles in the nanogel network [102,103]. Eventually, such a nanohybrid was also adapted to prepare a radiosensitizer agent, exploiting the presence of the gold nanoparticles in the polymer [104].

**Figure 4:**
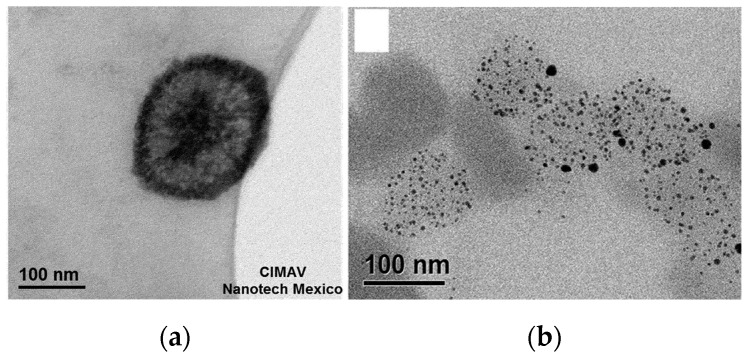
Transmission Electron Microcopy images of the nanogel: (**a**) The nanoparticle before the presence of Au, trough the staining with phosphotungstic acid is revealed in the core-structure (the further outer dark layer is attributed to initiator residues) and (**b**) after the in-situ generation of Au nanoparticles (without staining). Reprinted from Reference [76] by permission of the publisher (Taylor and Francis Ltd, http://www.tandfonline.com), Copyright 2017 Taylor & Francis Group.

A comparable system prepared by Claverie et al., embedded PEGylated acrylic nanogel with gold nanoparticle [76] and consolidates the previous results. In this case, authors evaluate the effect of various reaction parameters such as initiators, cross-linkers, monomers ratio, PEG concentration, and M_w_ on thermoresponsive responsive properties and on the nanogel structure. The dimensions of the prepared particles thus range from 52 to about 350 nm. Even if biological tests were not performed, the work also dedicates a large effort in investigating the core-shell structure of nanoparticles, evidencing the presence of PEG chains in the outer layer through techniques of differential scanning calorimetry and Transmission Electron Microcopy (TEM, Figure 4).

The inner structure of these particles is instead constituted by the diethylaminoethyl methacrylate polymer, confirming the description of Nagasaki [74]. Various PEG methacrylate in the M_w_ range of 500–4000 Da were selected to evaluate the effect of chain lengths; data show that, typically, shorter PEG chains bring to larger particles, and the shortest PEG chain (~ 500 Da) is indeed unable to stabilize the monomer droplets, yielding a non-uniform size distribution of nanoparticles. Additionally, various concentrations of the PEG monomer were tried, finding the main outcomes at the polymerization stage: Increasing the content of PEG acrylate decreases the size of the nanogel particle. This is reasonably due to the stabilizing effect of PEG chains towards smaller particles in the emulsion polymerization process. Gold nanoparticles were eventually inserted by in situ reduction of HAuCl_4_ to demonstrate the capability of the nanogel to host metal nanoparticles.

**Figure 5:**
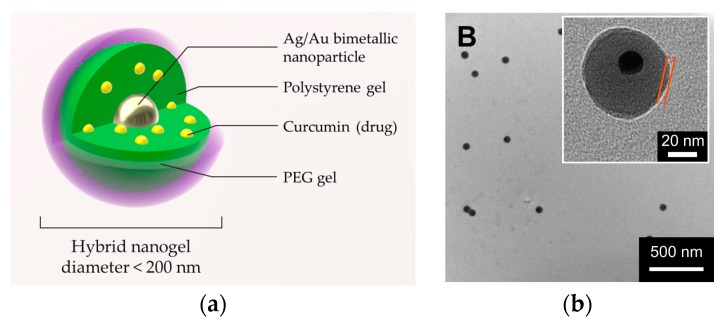
The hybrid nanogel prepared by Zhou [77]: (**a**) Schematic representation and (**b**) TEM image which evidenced the shell thickness. Image (**b**) reprinted from Reference [77] Copyright (2011), with permission from Elsevier.

Zhou et al. prepared a noteworthy example of core-shell PEGylated nanogel, with a nucleus of Au/Ag, a hydrophobic inner polymer, and a crosslinked PEG shell [77] (scheme in Figure 5). 

The inner layer consists of crosslinked styrene/divinylbenzene chains, and the nature of this polymer allowed for the hosting of a hydrophobic drug as curcumin. On the other hand, the PEG polymer of the outer layer provides stability to the system in a physiological environment and affords a volumetric phase transition temperature-dependent. The release of the drug, driven by polymer shrinkage, is thus triggered by the photothermal effect of the Au/Ag core nanoparticle. The strategy, adopted for the synthesis of this composite, involves two steps of precipitation polymerization: The first on the metallic water-dispersed nanoparticle, and the second, for the PEG decoration on the prepared nanoparticle of crosslinked polymer. The PEG network is obtained, adopting two methacrylate oligo(ethylene glycol) derivatives as comonomers and polyethylene glycol dimethacrylate as a crosslinker. The biocompatibility of the system is appreciable: In the absence of the drug viability of B16F10 cells remain unaltered for all the tested concentrations (up to 450 μg/mL). The therapeutic result is an efficient penetration in mouse melanoma cells B16F10 and a consequent cytotoxicity due to the thermal activated drug release, synergistic with the photothermal effect. 

**Figure 6:**
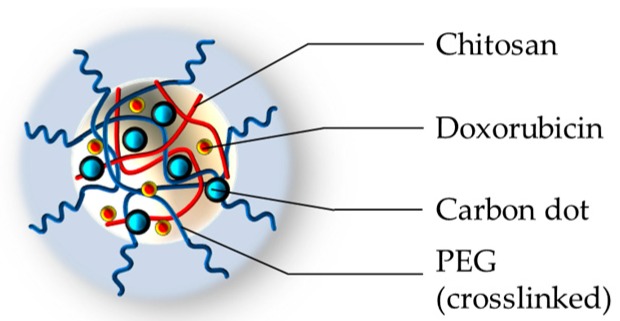
Graphic representation of PEG-chitosan-(carbon dots) hybrid nanogel [78].

Later, Zhou and coworkers developed an articulated example of biocompatible nanogel [78], prepared by adopting a conceptually different approach: The PEG chains have been crosslinked in the presence of chitosan, physically entangling this polymer in the PEG network. The adopted crosslinker and comonomers were PEG-dimethacrylate and oligoethylene glycol methacrylate, respectively. The nanogel has been employed to prepare a nanohybrid embedding carbon dots distributed in the structure; these provided a persistent fluorescence and a photothermal effect upon near infrared (NIR) irradiation. The final nanohybrid, represented in Figure 6, displays a mean diameter around 120 nm at pH 7.4. It has been loaded with an anticancer drug (doxorubicin), obtaining a thermal triggered release once irradiated with NIR light.

The nanogel also shows fluorescence, upon irradiation at 405 nm, due to the presence of incorporated carbon dots. The fluorescent behavior, the biocompatibility, and the cytotoxic efficacy have been tested by in vitro experiments on human prostate cancer cells DU145 (up to 100 μg/mL of nanohybrid) and in vivo on mice models to evaluate the possible animal toxicity. The permanence of the fluorescence was observed, and a strong decrease on the cellular viability was detected upon NIR irradiation of the drug-loaded system. The in vivo biocompatibility of the nanohybrid was proven by direct comparison between C57BL/6 mice injected with the drug free nanogel and non-injected animals. The histopathological data showed renal and hepatic tissues without signs of toxicity, confirming the in vitro results. The synergistic effect of doxorubicin and hyperthermia was also proved.

**Figure 7:**
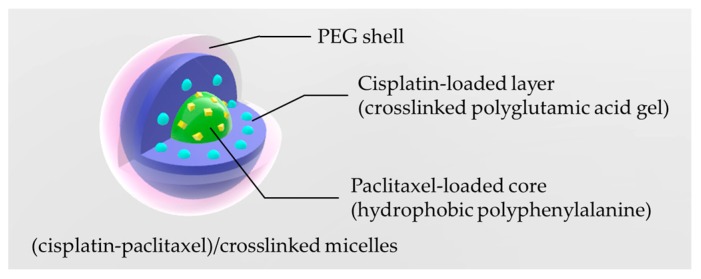
Structure of the nanogel micelles [30] with the expected core-layer-shell framework to host the different drugs.

Crosslinked polymeric micelles as system for the co-delivery of different drugs, have been prepared by Bronich and her research group [30]. This nanogel is composed by a block copolymer of polyethylene glycol-polyglutamic acid-polyphenylalanine that confers a hybrid structure core–layer–shell with PEG in the external zone (Figure 7). 

Nanogel is achieved by preparing the copolymer micelles in the aqueous phase, activation of glutamic groups by carbodiimide, and addition of ethylenediamine to carry out the crosslinking. The formed network, maintaining the PEG chains in the external shell, eventually stabilizes the micellar geometry. The nanogel is exploited for the co-delivery of cisplatin and paclitaxel, performing in vitro experiments on human ovarian A2780 cancer cells and in vivo tests on xenografts mice models. The nanogel micelles have proven to be non-toxic to the cells up to a concentration of 5 mg/mL, while the fully loaded system showed a remarkable cytotoxicity and a synergistic effect of the two drugs. It is worth noting how this effect occurs only when the two drugs are present together on the same nanogel particle. This assumption arises from comparative experiments performed incubating the cells in co-presence of paclitaxel and cisplatin, but with the drugs loaded on separate particles. The in vivo experiments confirmed the synergistic effect and showed higher accumulation of the drug in the tumoral tissue. Results also evidenced the absence of histopathological changes, respect to untreated animals, in the kidney tissue of animals treated with the nanogel. In particular, authors underline how the lower nephrotoxicity might represent a strong advancement concerning the use of this drug, considering how the renal toxicity constitutes the side effect that most restrict cisplatin therapies [105]. Another evidenced effect of this drug delivery system is the higher platinum accumulation in spleen and liver, although the value is greatly reduced after a month, and no signs of splenic or hepatic toxicity were detectable. Even still, through histopathologic analyses, the nanogel particles inside the spleen were only localized in the red pulp and poorly in lymphoid regions, suggesting the absence of free drug in these areas and providing a possible rationale for the absence of splenotoxicity.

## 3. Nanogels inside Liposomes: Nanolipogels

A quite different type of nanohybrids can be obtained combining nanogel particles with liposomes. In these structures, named nanolipogels, liposomes act as templates to host the nanogel in their internal compartment and remaining after polymerization as an external lipidic shell. As a further effect, the presence of the gel in the core imparts stability to the whole nanostructure. On these nanohybrids, a stealth strategy can be achieved simply preparing liposomes with PEGylated phospholipids. While polymers embedded in liposomes have been studied for some years [18], nanogels (i.e., crosslinked polymers) are only present in few cases. However, the various publications, herein described, show a positive combination of nanogels with stealth liposomes.

**Figure 8:**
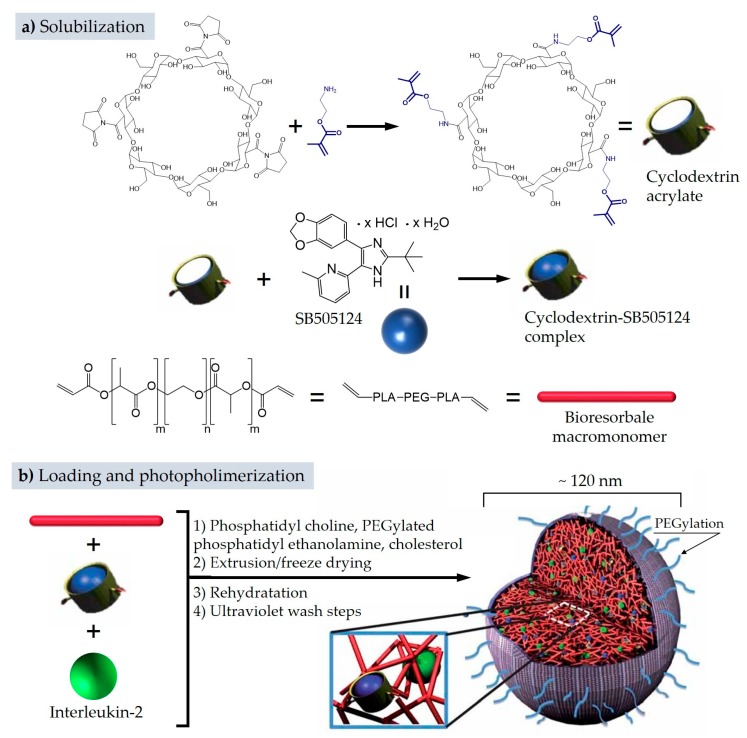
Overall structure of the nanolipogel with the different components and the preparation steps. The cyclodextrin-methacrylate is represented as a hollow cylinder that hosts the transforming growth factor-β inhibitor SB505124 (blue sphere). Adapted by permission from Springer Nature: Nature Materials [79], Copyright 2012.

An appreciable milestone of these nanohybrids may be represented by the work of Fahmy and colleagues [79], who have prepared and tested in vivo on a nanoscale liposomal polymeric gel that can release simultaneously hydrophilic and hydrophobic compounds. The liposomes were prepared, along with phosphatidylcholine and cholesterol, with an aliquot of PEGylated phosphatidylethanolamine (M_w_ of PEG = 2000 Da) in order to present a stealth surface; the final structure show a diameter of 120 nm and a near zero zeta potential. The inner nanogel is based on a bisacrylate-terminated PEG–lactide bioresorbable macromonomer (M_w_ ~ 4000 Da) polymerized via an UV-activated initiator (Figure 8).

**Figure 9:**
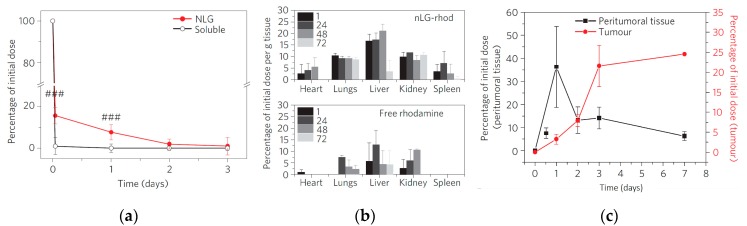
In vivo biodistribution data: (**a**) Persistence in the blood stream of free and loaded rhodamine (### denotes P < 0.01 in the two population t-test); (**b**) cumulative biodistribution of free and loaded rhodamine (the legend denotes the post-injection hours); and (**c**) accumulation of rhodamine (delivered by the nanolipogel) in the tumoral tissue and residual amount in the peritumoral region. Error bars in all plots represent ± std. dev. Reprinted by permission from Springer Nature: Nature Materials [79], Copyright 2012.

The incorporation of the nanogel involves the reconstitution of lyophilized liposomes in a precursor solution containing acrylate macromonomers and initiator. The suspension is then diluted five times to avoid massive gelation outside the liposomes and eventually exposed to UV light for the nanogel curing. The loading of the drugs into nanogel occurs by introducing in the precursor solution of the two substances: Transforming growth factor-β inhibitor (SB505124, Figure 8, commercially available, denoted SB) and interleukin-2 (a 15 kDa signaling protein belonging to the immune system [106]). These two compounds were chosen specifically for their individual pharmacological properties, to assess the synergistic anticancer effect by increasing the activity of natural killer cells. The molecule SB, for an optimal encapsulation and a sustained release, has been previously inserted in a cyclodextrin derivative bearing acrylic groups (Figure 8 together with the complete structure). The overall outcome of this nanohybrid was thus the simultaneous and localized release of the two drugs. Since numerous pharmacokinetic data of this PEGylated structure have been collected, we deemed appropriate to detail the main outcomes. In vivo experiments on murine models monitored the biodistribution, safety, and toxicological profile of the fully laden platform as well as of the empty system, adopting animals administered with PBS as control. These have provided useful information on circulation lifetime, possible secondary effects, and accumulation sites. Specific tests were also performed to evaluate the passive infiltration capability of this delivery system inside the tumoral microenvironment with respect to peritumoral tissue. Acute toxicity was evaluated seven days after a single intravenous dose of the nanolipogel: The blood parameters showed values within normal physiological ranges, absence of detectable hepatotoxicity, absence of renal toxicity, and absence of hematic toxicity. Lungs were screened by histological analysis revealing no signs of pulmonary toxicity. In addition, markers for inflammatory responses revealed no statistical differences with respect to control animals administered with phosphate buffered solution (PBS). Biodistribution, pursued by loading the nanolipogel with rhodamine, displayed an increased persistence in the blood stream, with a 24 h post-injection concentration of about 8% of the initial dose. The residual rhodamine at 1 h post-injection time is no more detectable, while the residual nanolipogel at the same time lapse is ~16% (Figure 9a). The analysis of organs showed the primary accumulation of rhodamine in liver, kidney, and lungs (Figure 9b), with a higher level for the encapsulated substance. The mediated delivery, valued after an injection nearby the tumor microenvironment, revealed a sustained release of rhodamine, with a migration from the peritumoral area to the tumoral mass, and a final accumulation in the inner region of around 25% of the administered dose (Figure 9c).

The therapeutic effects were analyzed on B16/B6 murine melanoma model: Experiments were performed on single tumors and on metastatic ones, respectively, managed with local and systemic treatment; achievements were consistent with the pharmacokinetics data.

Another relevant example from the same research group presents the preparation of nanolipogels for the treatment of lupus erythematosus [80]. The average diameter of the particles is 225 nm and their structure is analogous to the previously reviewed. The essential difference lay in the drug loaded: Mycophenolic acid (MPA) as immunosuppressant. However, in addition to the efficacy trials on NZB/W F1 mice, the drug delivery system has been tested on C57BL/6 murine models (not susceptible to lupus) to evaluate the hematological and organ toxicity; as the control were adopted animals administered with PBS. The animals were exposed to four daily doses of nanolipogels MPA loaded and the analyses were carried out 4, 7, and 14 days later. Clinical parameters related to renal function, hepatic function, and the complete blood counts presented all values within the reference ranges. An enhanced accumulation in the kidneys was detected for the nanogel, but again, no signs of renal or hepatic toxicity were detectable. As an additional note on the results, the treatment of lupus erythematosus by this nanohybrid carrying 5–azacytidine showed an encouraging regression of the disease [107].

**Figure 10:**
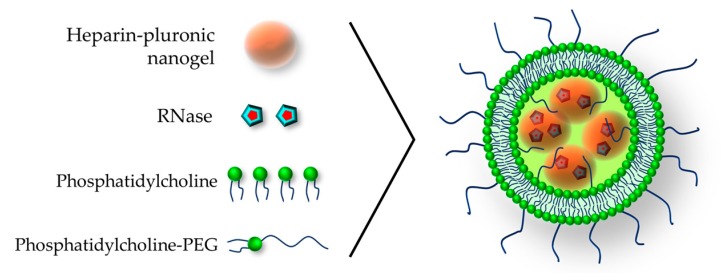
The PEGylated nanolipogel containing heparin-pluronic nanogel loaded with RNase. Adapted by permission from Springer Nature: Macromolecular Research [81], Copyright 2015.

A further contribution is the work of Ki Dong and colleagues, who developed PEGylated liposomes containing a heparin-pluronic physical nanogel for the delivery of ribonuclease A [81]. The liposomes size ranges from 200 to 300 nm and their preparation involve phosphatidylcholine PEGylated with chains of 4000 Da. The peculiarity of this work is the easy nanolipogel preparation; the whole procedure is indeed quite simple and driven by self-assembly. The liposome reconstitution from dry phospholipids and cholesterol occurs by sonication in an aqueous solution that already contains ribonuclease and the heparin–pluronic previously prepared nanogel (Figure 10). Subsequently, the material that was not encapsulated is removed by centrifugation. It is worth citing that authors describe in detail the synthesis of PEGylated phosphatidylcholine. 

The nanohybrid was tested in vitro on mouse fibroblasts NIH3T3, showing the drug delivery effect and the non-toxicity of the system, although the experiments were performed at a single concentration of 100 µg/mL.

**Figure 11:**
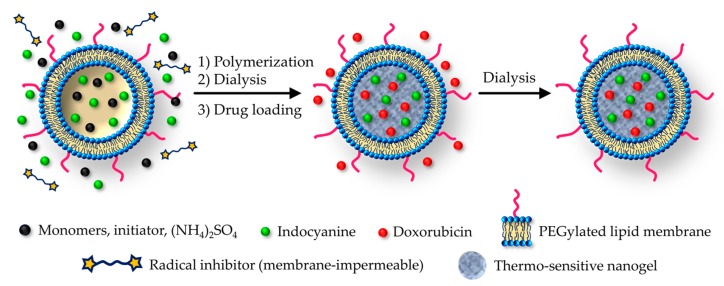
The nanolipogel preparation strategy with the inhibition of polymerization outside the vesicle. Note: the radical inhibitor is a TEMPO-PEG-TEMPO molecule. Adapted with permission from Reference [82]. Copyright (2018) American Chemical Society.

In 2018, Zhang et al. prepared various liposome-camouflaged nanogels [82,108,109], adopting for all systems the PEGylation strategy with chains of 2000 Da grafted on the lipidic shell. Their most distinguished work [82] reports the drug delivery combined with the photothermal effect. In this case, the embedded nanogel is a typical thermoresponsive isopropyl-acrylamide polymer, inserted in the lipid vesicle through a quite sophisticated technique. As a first step, the liposomes were assembled in the aqueous gel precursor solution, together with monomers and radical initiator. Then, once the vesicles have been formed, a membrane-impermeable radical inhibitor is added and the polymerization is carried out (Figure 11); however, the nanogel is formed only inside liposomes, where the reaction is not inhibited. 

**Figure 12:**
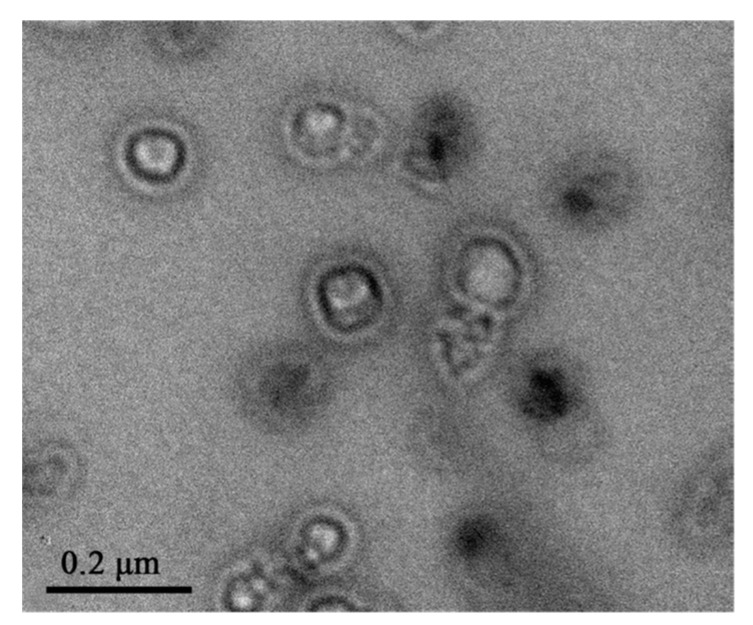
TEM image of the nanolipogel prepared by Zhang and coworkers. Reprinted with permission from Reference [82]. Copyright (2018) American Chemical Society.

The photo-active substance, indocyanine green, is added in the precursor solution and is actively encapsulated by liposome closure, while doxorubicin was introduced successively through the ammonium sulfate gradient method. The structure presents an overall mean diameter of 106 nm at 25 °C (TEM micrograph in Figure 12). This drug delivery system was tested on 4T1 murine breast cancer cells and on human red blood cells. Biological tests revealed a complete hemocompatibility at 100 μg/mL, as well as a full cellular compatibility up to 1000 μg/mL.

In a slight variation proposed by the same authors [109], the nanogel was prepared by adopting cysteine dimethacrylate as redox responsive disulfide crosslinker. Thus, the nanohybrid release is triggered by the presence of glutathione inside the cells. The other components of the structure have been left substantially unchanged and the in vitro biocompatibility/hemocompatibility were unaffected by this variation.

## 4. Zwitterionic Nanogels

A different and relatively recent technique to extend the circulating half-life of nanosystems is represented by the decoration with zwitterionic structures. This type of functionalization does not present specific synthetic difficulties and may provide a valuable alternative to the strategy of PEGylation [110], even on hybrid nanogel, as disclosed by the positive outcomes herein collected. The idea is based on the notable antifouling properties of zwitterionic surfaces [111], which also reproduces a direct imitation of the character of cell membranes. The presence of both positive and negative charges close to each other along the whole structure lead to a hydrophilic and strongly polar surface, consequently, a stable layer of water molecules is created around the nanoparticle. This aqueous layer is firmly structured and reveals a high efficiency in preventing the non-specific protein adsorption [112]. Moreover, the zwitterionic structure maintains a net charge balanced with a zeta potential near to zero, limiting the electrostatic interactions with proteins [113]. 

**Figure 13:**
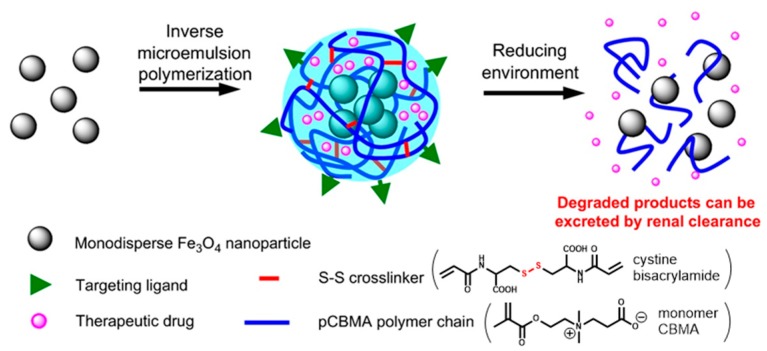
Schematic passages for the preparation of zwitterionic hybrid nanogel. Reprinted from Reference [83], Copyright (2011), with permission from Elsevier.

One of the first applications of such strategy to hybrid nanogels has been achieved by Jiang and colleagues in 2011 [83]. Their work describes the encapsulation of Fe_3_O_4_ magnetic nanoparticles in a nanogel based on carboxybetaine methacrylate (CBMA) and cystine bisacrylamide as a reduction–sensitive crosslinker (Figure 13). Besides, through the acid groups of carboxybetaine, the nanohybrid was further decorated with a RGD peptide to increase the uptake by target cells. As a model drug was inserted as a fluorescein-labeled dextran, it was easily traceable and commercially available. The release experiments in the presence of dithiothreitol revealed an expulsion of about 80% of the fluorescent load within 24 h, while in the absence of the reducing agent, a release lower than 5% was observed at the same time lapse. The degradation of the nanogel was also confirmed by the magnetic resonance imaging experiments and DLS analyses. These showed the nanogel particles of ~110 nm disappear after the addition of dithiothreitol, to be replaced by nanoparticles of ~10 nm, the diameter of the previously embedded Fe_3_O_4_ particles. Biological tests were performed on macrophages and on HUVEC cells, reporting in both cell lines, no observable cytotoxicity at the concentrations of 1–30 ppm of iron content, although the iron content per mg of nanogel was reported. Authors further mention that, after lyophilization, the product was redispersed in PBS obtaining a reconstituted nanogel with an unaltered dimension (110 nm).

One more work of Jiang combine the zwitterionic feature with a singular aspect, the “softness” of the particles, to produce a nanogel with a longer circulating time and lower splenic accumulation [84]. The study of biomechanical properties of the nanoparticles, as a leading aspect of their physiological behavior is indeed another relevant issue, recently highlighted by noteworthy works [47,114]. While it concerns an aside topic, it is worthwhile to simply recall the direct linkage between elastic module and circulating time of erythrocytes [115], as well as the example of a microgels mimicking the red blood cells rheology to acquire an extended half-life [116].

**Figure 14:**
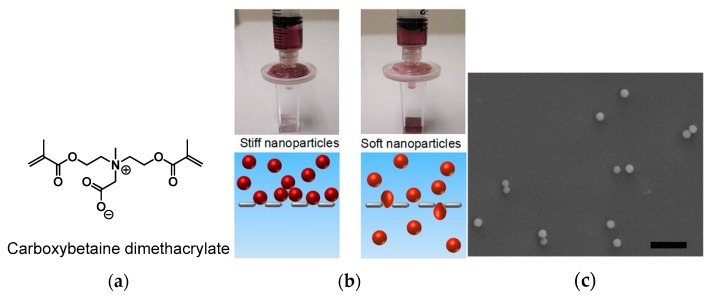
Zwitterionic stiff/soft nanogel: (**a**) Structure of the zwitterionic crosslinker; (**b**) a simple filtration reveal the qualitatively different behavior of the two nanogels; and (**c**) scanning electron microscopy image of the soft nanogel particles (scale bar = 1 μm). Reprinted with permission from Reference [84]. Copyright 2012 American Chemical Society.

In the described case [84], the authors prepared in different parallel nanogels, all carrying gold nanoparticles as a core and poly(carboxybetaine) as a polymer, but containing different amounts of the crosslinker carboxybetaine dimethacrylate (Figure 14a). This crosslinker, together with the monomer, ensures the complete zwitterionic nature of the nanogel. The various batches of nanohybrids share the same diameter, around 120 nm; however, possessing different stiffness, they provide different biological outcomes. The in vivo tests on animal models indicate the direct correlation between higher crosslinker density and longer circulation half-life, confirming the proposed strategy. A simple assay, which is still immediate, has been obtained by filtering the nanogel particles through a 0.22 µm syringe filter (Figure 14b), although the hydrodynamic diameters were the same, the response to filtration was very different.

The biodistribution showed that almost all nanoparticles accumulated in the liver and spleen with minor accumulation in the other organs. However, the splenic accumulation decrease substantially at each decrement of the crosslinker in the polymer, along with an increased circulating half-life. Moreover, even if on a smaller scale, the increase of gold concentration in the blood as the crosslinker decrease is visible, confirming the previous result. In these experiments, the control was not necessary since the data derived from the quantification of gold was not naturally present in the target organs. Additional proofs of the stealth behavior have been obtained, incubating macrophage cells with the nanogel and with bare gold nanoparticles (nanogel concentration: 42 μg/mL, corresponding to 5 ppm Au), evidencing the efficacy of the zwitterionic compound in eluding the uptake by macrophages.

**Figure 15:**
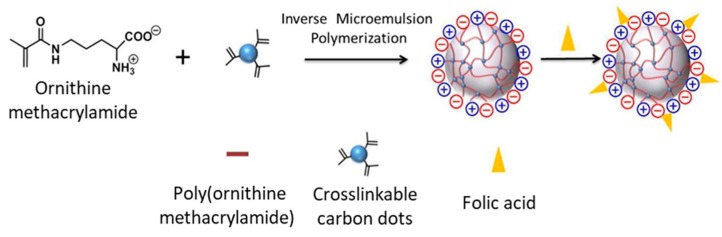
Monomer structure and zwitterionic nanogel-carbon dots assembly. Reprinted from Reference [85], Copyright (2016), with permission from Elsevier.

Another type of nanoparticle efficiently involved in a zwitterionic nanogel is represented by carbon dots. To preserve the loading capacity of the nanogel and obtain a stable encapsulation, Liu and coworkers [85] have functionalized these nanoparticles with methacrylate groups. Through this strategy, they prepared a nanogel interlaced with carbon dots, turning these smaller inorganic particles to be an integral part of the polymer network, retaining both their fluorescence and the features of the gel. The zwitterionic character is provided by ornithine methacrylamide (Figure 15), a non-proteinogenic amino acid derivative chosen for its antifouling properties [117]. Finally, the external surface of the nanohybrid was decorated with folic acid as a targeting ligand. The payload was a fluorescent labeled dextran (10 kDa), adopted as a model to study the loading and the releasing profile of a hydrophilic drug, such as peptides or nucleic acids.

Different quantities of the crosslinker (carbon dots), from 0.5% to 10% (*w*/*w*), have been tested resulting in nanoparticles of different diameters, from near 170 to 114 nm. This effect is ascribable to two factors: The higher entangling degree, which lead to a lower gel swelling [118,119], and the presence of multiple nucleation centers during the polymerization, resulting in the formation of smaller nanoparticles [120]. In the present study [85], authors used the smallest particles for all the experiments, rationalizing the choice on the basis of their optimal size [121]. As an experiment to validate the interactions with proteins and general stability in physiological media, the nanohybrid was incubated for 24 h in 5% human plasma fibrinogen or in 5% bovine serum albumin solutions with PBS, not revealing in either case an increase of the hydrodynamic size. In addition, in vitro tests on NIH/3T3 fibroblasts revealed a good biocompatibility of the structure; the cell viability was around 90% after 24 h of incubation at a concentration 1 mg/mL. Further biological tests were also performed on SKOV3 ovarian cancer cells to prove the targeting effect of the folic acid decoration on cellular uptake.

## 5. Challenges and New Perspectives for Hybrid Nanogels

Nanogels are 3D constructs in colloid form with a crosslinked polymeric scaffold, typically stable in aqueous media. Due to the high complexity of these systems and to the high need of reproducible devices, a proper understanding of these structures is fundamental [122,123]. Respect to other nanosystems, the strength of hybrid nanogels is their ability to improve circulating lifetimes and bioavailability of the carried drugs, joined with a wide versatility of the structures. The capability of nanogels to be variously decorated with molecules and polymeric chains, their elastic structure, their possible zwitterionic surface, and the possibility to be easily combined with other nanostructures, make them a very suitable candidate to overcome the current limitations of many drug delivery systems. Possible weaknesses are still present, a price for their versatility may be identified in the polymerization, the synthetic step required for their preparation, absent for commercially available inorganic or metal nanostructures and pivotal to obtain a proper nanohybrid. Besides, compared to “hard” scaffolds like carbonaceous nanomaterials, their storage may require delicate steps, such as the lyophilization, to prepare a stable and reconstitutable product. 

Concerning their medical application, nanogels are still present in clinical trials, mainly focused on cholesterol–pullulan structures for cancer immunotherapy [124,125,126,127,128]. However, many challenges still exist in order to widen their clinical development. First of all, the mechanisms and the pathways of active intracellular uptake should be deeply investigated in order to understand their fate in living organisms. The quick clearance by the immune system represents a classic problem for drug delivery scaffolds, together with low selectivity in cell targeting and low efficiency to overcome biological hurdles and barriers, such as the blood-brain barrier [71,129]. For this purpose, the easy traceability and the adaptable properties of hybrid nanogels would bring a worthwhile contribution in the development of experiments devised to acquire a more accurate knowledge of the biological mechanisms involved. As observed, the tunable softness, the bioresorbable structures, and the different types of possible decoration are features commonly achievable by nanogels, which may represent key variables for understanding the biological responses.

In addition, safety concerns should also be taken into account. Nanotoxicity in the last years is working in parallel with nanomedicine in the aim of understand the possible negative impact between nano-objects and biological systems. Preliminary studies have actually indicated that, in many cases, nanosystems contributed to inflammation, damage, and unwanted cell uptake [130,131]. In this respect, although several efforts should still be involved to understand the factors behind toxicity, we here observed many strategies conceived by nanogels to minimize these drawbacks.

The reproducible and consistent manufacturing of nanosystems is another relevant question that should be solved. It is commonly known that the scale up of nano-objects production represent a considerable challenge since, by increasing the scale, the formation of novel surface is less-favored respect to the bulk. Moreover, given the presence of multiple reaction conditions, as the use of organic solvents, emulsification, ultrasound and homogenization, is fundamental to the identification of the main parameters to obtain reproducible, storable (e.g., upon freeze-drying), and fully reconstitutable nanogels. High attention should be paid to the reproducibility of the process to ensure that the characteristics of the colloid remain the same during the scale up procedure, avoiding instability or aggregation before use. Ultimately, the sterilization methods may also influence the characteristics of the product, affecting mechanical properties and degradation [132,133], and from the perspective of clinical application should be adequately considered.

## Figures and Tables

**Table 1:** The main morphologies of the described nanogels with summarized the biological outcomes.

Schematized Structure of the Hybrid	Stealth Strategy	Size	Biological Results	Reference
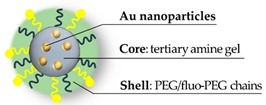	PEG chains (~2400 Da, 7200 Da ^1^)	80 nm ^a^	In vitro: no toxicity on HuH-7 (50 μg/mL), HeLa (480 µg/mL) ^1^	[74,75]
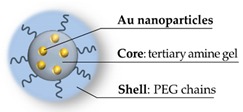	PEG shell (500–4000 Da)	52–350 nm ^a^	(see note ^2^)	[76]
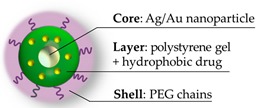	PEG gel (300 Da), PEG shell (550 Da)	25–40 nm ^a^ [40–60 ^b^]	In vitro: no toxicity on B16F10 mouse melanoma cells (450 μg/mL)	[77]
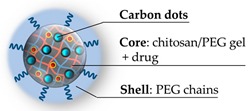	PEG/chitosan gel, PEG chains (550 Da)	120 nm ^a^ (pH 7.4) [180 nm ^b^]	In vitro: no toxicity on DU145 cells (100 μg/mL) In vivo: C57BL/6 mice models, histology: no signs of toxicity on liver and kidney.	[78]
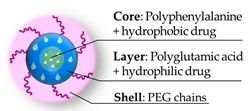	PEG shell (5000 Da)	90 nm ^a^ (pH 7.4)	In vitro: no toxicity on A2780 cells (5 mg/mL) In vivo: xenograft mice, histology: no alteration on kidney tissue, no signs of splenic or hepatic toxicity, most platinum accumulated eliminated after 1 month.	[30]
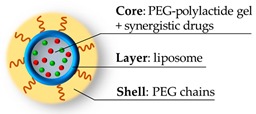	PEGylated liposome (2000 Da), bioresorbable gel	120 nm ^a^	In vitro: only release tests in phosphate buffer (pH 7.4) In vivo: B16/B6 mice models: absence of renal and hepatic toxicity. Blood values in physiological ranges, no signs of pulmonary toxicity. No inflammatory response markers. Extended circulation lifetime of carried drug, improved biodistribution.	[79]
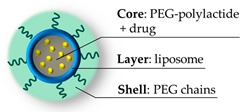	PEGylated liposome (2000 Da), bioresorbable gel	225 nm ^a^	In vitro: only internalization tests on CD4 T cells In vivo: C57BL/6 mice models: no hepatic, hematological and general organ toxicity; repeated doses and analyses show values in physiological ranges: complete blood counts, renal and hepatic functions. Protect from nephritis.	[80]
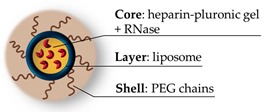	PEGylated liposome (4000 Da), biocompatible gel	300 nm ^a^	In vitro: no toxicity on NIH3T3 mouse fibroblasts (100 μg/mL)	[81]
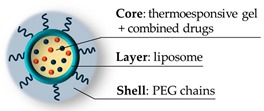	PEGylated liposome (2000 Da)	106 nm ^a^ [103 nm ^b^]	In vitro: no toxicity on 4T1 murine cancer cells (1000 μg/mL), hemocompatibility on human blood (100 μg/mL)	[82]
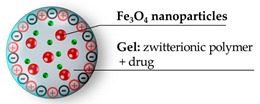	Zwitterionic polymer: carboxybetaine, reduction-sensitive crosslinker	110 nm ^a^	In vitro: no cytotoxicity on macrophages (RAW264.7) and HUVEC cells (iron content: 30 ppm) ^3^	[83]
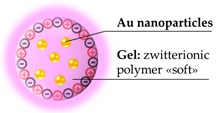	Zwitterionic polymer: carboxybetaine, softness of the structure	120 nm ^a^	In vitro: no uptake from macrophage cells (5 ppm of Au, corresponding to 42 μg/mL of nanogel) In vivo: Sprague Dawley rats: biodistribution study, most accumulation in liver and spleen. Increasing softness extend the circulation half-life and reduces splenic accumulation	[84]
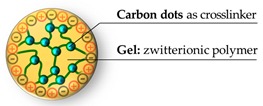	Zwitterionic polymer: ornithine	114 nm ^a^	In vitro: 90% viability on NIH/3T3 fibroblasts (1000 μg/mL), no protein bonded after incubation in protein solution	[85]

^1^ Polyethylene glycol chains of 7200 Da and toxicity on HeLa are referred to in Reference [75]. ^2^ Even in the absence of biological data, this work has been considered relevant for the reported characterization data. ^3^ Authors do not state the iron content per mg of nanogel. ^a^ Measured by dynamic light scattering. ^b^ Measured by electronic microscopy.

## References

[1] Oh J.K., Drumright R., Siegwart D.J., Matyjaszewski K. (2008). The development of microgels/nanogels for drug delivery applications. Prog. Polym. Sci..

[2] Karimi M., Ghasemi A., Sahandi Zangabad P., Rahighi R., Moosavi Basri S.M., Mirshekari H., Amiri M., Shafaei Pishabad Z., Aslani A., Bozorgomid M. (2016). Smart micro/nanoparticles in stimulus-responsive drug/gene delivery systems. Chem. Soc. Rev..

[3] Qian Z.Y., Fu S.Z., Feng S.S. (2013). Nanohydrogels as a prospective member of the nanomedicine family. Nanomedicine.

[4] Peng H.S., Chiu D.T. (2015). Soft fluorescent nanomaterials for biological and biomedical imaging. Chem. Soc. Rev..

[5] Hamidi M., Azadi A., Rafiei P. (2008). Hydrogel nanoparticles in drug delivery. Adv. Drug Deliv. Rev..

[6] Zhang H., Zhai Y., Wang J., Zhai G. (2016). New progress and prospects: The application of nanogel in drug delivery. Mater. Sci. Eng. C.

[7] Eckmann D.M., Composto R.J., Tsourkas A., Muzykantov V.R. (2014). Nanogel carrier design for targeted drug delivery. J. Mater. Chem. B.

[8] Jiang Y., Chen J., Deng C., Suuronen E.J., Zhong Z. (2014). Click hydrogels, microgels and nanogels: Emerging platforms for drug delivery and tissue engineering. Biomaterials.

[9] Oh J.K., Lee D.I., Park J.M. (2009). Biopolymer-based microgels/nanogels for drug delivery applications. Prog. Polym. Sci..

[10] Chacko R.T., Ventura J., Zhuang J., Thayumanavan S. (2012). Polymer nanogels: A versatile nanoscopic drug delivery platform. Adv. Drug Deliv. Rev..

[11] Tang Z., He C., Tian H., Ding J., Hsiao B.S., Chu B., Chen X. (2016). Polymeric nanostructured materials for biomedical applications. Prog. Polym. Sci..

[12] Kabanov A.V., Vinogradov S.V. (2009). Nanogels as pharmaceutical carriers: Finite networks of infinite capabilities. Angew. Chemie Int. Ed..

[13] Wu H.Q., Wang C.C. (2016). Biodegradable smart nanogels: A new platform for targeting drug delivery and biomedical diagnostics. Langmuir.

[14] Karg M. (2016). Functional Materials Design through Hydrogel Encapsulation of Inorganic Nanoparticles: Recent Developments and Challenges. Macromol. Chem. Phys..

[15] Molina M., Asadian-Birjand M., Balach J., Bergueiro J., Miceli E., Calderón M. (2015). Stimuli-responsive nanogel composites and their application in nanomedicine. Chem. Soc. Rev..

[16] Nguyen M., Felidj N., Mangeney C. (2016). Looking for Synergies in Molecular Plasmonics through Hybrid Thermoresponsive Nanostructures. Chem. Mater..

[17] Wang H., Chen Q., Zhou S. (2018). Carbon-based hybrid nanogels: A synergistic nanoplatform for combined biosensing, bioimaging, and responsive drug delivery. Chem. Soc. Rev..

[18] Raemdonck K., Braeckmans K., Demeester J., De Smedt S.C. (2014). Merging the best of both worlds: Hybrid lipid-enveloped matrix nanocomposites in drug delivery. Chem. Soc. Rev..

[19] Sierra-Martin B., Fernandez-Barbero A. (2015). Multifunctional hybrid nanogels for theranostic applications. Soft Matter.

[20] Rejinold N.S., Jayakumar R., Kim Y.C. (2015). Radio frequency responsive nano-biomaterials for cancer therapy. J. Control. Release.

[21] Li Z., Ye E., David, Lakshminarayanan R., Loh X.J. (2016). Recent Advances of Using Hybrid Nanocarriers in Remotely Controlled Therapeutic Delivery. Small.

[22] Mai B.T., Fernandes S., Balakrishnan P.B., Pellegrino T. (2018). Nanosystems Based on Magnetic Nanoparticles and Thermo- or pH-Responsive Polymers: An Update and Future Perspectives. Acc. Chem. Res..

[23] Macchione M.A., Biglione C., Strumia M. (2018). Design, synthesis and architectures of hybrid nanomaterials for therapy and diagnosis applications. Polymers (Basel).

[24] Kowalczuk A., Trzcinska R., Trzebicka B., Müller A.H.E., Dworak A., Tsvetanov C.B. (2014). Loading of polymer nanocarriers: Factors, mechanisms and applications Dedicated to Prof. Stanislaw Penczek on the occasion of his 80th birthday. Prog. Polym. Sci..

[25] Cayre O.J., Chagneux N., Biggs S. (2011). Stimulus responsive core-shell nanoparticles: Synthesis and applications of polymer based aqueous systems. Soft Matter.

[26] Plamper F.A., Richtering W. (2017). Functional Microgels and Microgel Systems. Acc. Chem. Res..

[27] Panday R., Poudel A.J., Li X., Adhikari M., Ullah M.W., Yang G. (2018). Amphiphilic core-shell nanoparticles: Synthesis, biophysical properties, and applications. Colloids Surf. B Biointerfaces.

[28] Hasegawa U., Nomura S.I.M., Kaul S.C., Hirano T., Akiyoshi K. (2005). Nanogel-quantum dot hybrid nanoparticles for live cell imaging. Biochem. Biophys. Res. Commun..

[29] Liu J., Detrembleur C., Mornet S., Jérôme C., Duguet E. (2015). Design of hybrid nanovehicles for remotely triggered drug release: An overview. J. Mater. Chem. B.

[30] Desale S.S., Cohen S.M., Zhao Y., Kabanov A.V., Bronich T.K. (2013). Biodegradable hybrid polymer micelles for combination drug therapy in ovarian cancer. J. Control. Release.

[31] Ekkelenkamp A.E., Elzes M.R., Engbersen J.F.J., Paulusse J.M.J. (2018). Responsive crosslinked polymer nanogels for imaging and therapeutics delivery. J. Mater. Chem. B.

[32] Hood M.A., Mari M., Muñoz-Espí R. (2014). Synthetic strategies in the preparation of polymer/inorganic hybrid nanoparticles. Materials (Basel).

[33] Mavila S., Eivgi O., Berkovich I., Lemcoff N.G. (2016). Intramolecular Cross-Linking Methodologies for the Synthesis of Polymer Nanoparticles. Chem. Rev..

[34] Landfester K., Musyanovych A. (2010). Hydrogels in miniemulsions. Chemical Design of Responsive Microgels.

[35] Cao Z., Ziener U. (2013). Synthesis of nanostructured materials in inverse miniemulsions and their applications. Nanoscale.

[36] Oh J.K., Bencherif S.A., Matyjaszewski K. (2009). Atom transfer radical polymerization in inverse miniemulsion: A versatile route toward preparation and functionalization of microgels/nanogels for targeted drug delivery applications. Polymer (Guildf).

[37] Sanson N., Rieger J. (2010). Synthesis of nanogels/microgels by conventional and controlled radical crosslinking copolymerization. Polym. Chem..

[38] Siegwart D.J., Oh J.K., Matyjaszewski K. (2012). ATRP in the design of functional materials for biomedical applications. Prog. Polym. Sci..

[39] Fan M., Wang F., Wang C. (2018). Reflux Precipitation Polymerization: A New Platform for the Preparation of Uniform Polymeric Nanogels for Biomedical Applications. Macromol. Biosci..

[40] Cortez-Lemus N.A., Licea-Claverie A. (2016). Poly(N-vinylcaprolactam), a comprehensive review on a thermoresponsive polymer becoming popular. Prog. Polym. Sci..

[41] Motornov M., Roiter Y., Tokarev I., Minko S. (2010). Stimuli-responsive nanoparticles, nanogels and capsules for integrated multifunctional intelligent systems. Prog. Polym. Sci..

[42] Seo M., Beck B.J., Paulusse J.M.J., Hawker C.J., Kim S.Y. (2008). Polymeric nanoparticles via noncovalent cross-linking of linear chains. Macromolecules.

[43] Cao R., Gu Z., Hsu L., Patterson G.D., Armitage B.A. (2003). Synthesis and characterization of thermoreversible biopolymer microgels based on hydrogen bonded nucleobase pairing. J. Am. Chem. Soc..

[44] Chen Y., Ballard N., Bon S.A.F. (2013). Waterborne polymer nanogels non-covalently crosslinked by multiple hydrogen bond arrays. Polym. Chem..

[45] Ramos J., Forcada J., Hidalgo-Alvarez R. (2014). Cationic Polymer Nanoparticles and Nanogels: From Synthesis to Biotechnological Applications. Chem. Rev..

[46] Acar H., Ting J.M., Srivastava S., LaBelle J.L., Tirrell M.V. (2017). Molecular engineering solutions for therapeutic peptide delivery. Chem. Soc. Rev..

[47] Guo P., Liu D., Subramanyam K., Wang B., Yang J., Huang J., Auguste D.T., Moses M.A. (2018). Nanoparticle elasticity directs tumor uptake. Nat. Commun..

[48] Sasaki Y., Akiyoshi K. (2010). Nanogel engineering for new nanobiomaterials: From chaperoning engineering to biomedical applications. Chem. Rec..

[49] Moshe H., Davizon Y., Menaker Raskin M., Sosnik A. (2017). Novel poly(vinyl alcohol)-based amphiphilic nanogels by non-covalent boric acid crosslinking of polymeric micelles. Biomater. Sci..

[50] Wang S., Ha Y., Huang X., Chin B., Sim W., Chen R. (2018). A New Strategy for Intestinal Drug Delivery via pH-Responsive and Membrane-Active Nanogels. ACS Appl. Mater. Interfaces.

[51] Soni K.S., Desale S.S., Bronich T.K. (2016). Nanogels: An overview of properties, biomedical applications and obstacles to clinical translation. J. Control. Release.

[52] Bertrand N., Grenier P., Mahmoudi M., Lima E.M., Appel E.A., Dormont F., Lim J.M., Karnik R., Langer R., Farokhzad O.C. (2017). Mechanistic understanding of in vivo protein corona formation on polymeric nanoparticles and impact on pharmacokinetics. Nat. Commun..

[53] Vicario-de-la-Torre M., Forcada J. (2017). The Potential of Stimuli-Responsive Nanogels in Drug and Active Molecule Delivery for Targeted Therapy. Gels.

[54] Allen T.M. (2004). Drug Delivery Systems: Entering the Mainstream. Science.

[55] Brigger I., Dubernet C., Couvreur P. (2002). Nanoparticles in cancer therapy and diagnosis. Adv. Drug Deliv. Rev..

[56] Gamucci O., Bertero A., Gagliardi M., Bardi G. (2014). Biomedical Nanoparticles: Overview of Their Surface Immune-Compatibility. Coatings.

[57] Lima A.C., Alvarez-Lorenzo C., Mano J.F. (2016). Design Advances in Particulate Systems for Biomedical Applications. Adv. Healthc. Mater..

[58] Ke P.C., Lin S., Parak W.J., Davis T.P., Caruso F. (2017). A Decade of the Protein Corona. ACS Nano.

[59] Monopoli M.P., Åberg C., Salvati A., Dawson K.A. (2012). Biomolecular coronas provide the biological identity of nanosized materials. Nat. Nanotechnol..

[60] Treuel L., Nienhaus G.U. (2012). Toward a molecular understanding of nanoparticle-protein interactions. Biophys. Rev..

[61] Hu C.-M.J., Zhang L., Aryal S., Cheung C., Fang R.H., Zhang L. (2011). Erythrocyte membrane-camouflaged polymeric nanoparticles as a biomimetic delivery platform. Proc. Natl. Acad. Sci. USA.

[62] Longmire M., Choyke P.L., Kobayashi H. (2008). Clearance properties of nano-sized particles and molecules as imaging agents: considerations and caveats. Nanomedicine.

[63] Tenzer S., Docter D., Rosfa S., Wlodarski A., Rekik A., Knauer S.K., Bantz C., Nawroth T., Bier C., Sirirattanapan J. (2011). Nanoparticle Size Is a Critical Physico- chemicalDeterminantoftheHumanBlood Plasma Corona: A Comprehensive Quantitative Proteomic Analysis. ACS Nano.

[64] Alexis F., Pridgen E., Molnar L.K., Farokhzad O.C. (2008). Factors Affecting the Clearance and Biodistribution of Polymeric Nanoparticles. Mol. Pharm..

[65] Papasani M.R., Wang G., Hill R.A. (2012). Gold nanoparticles: The importance of physiological principles to devise strategies for targeted drug delivery. Nanomed. Nanotechnol. Biol. Med..

[66] Yu M., Zheng J. (2015). Clearance Pathways and Tumor Targeting of Imaging Nanoparticles. ACS Nano.

[67] Gustafson H.H., Holt-Casper D., Grainger D.W., Ghandehari H. (2015). Nanoparticle uptake: The phagocyte problem. Nano Today.

[68] Dai Q., Wilhelm S., Ding D., Syed A.M., Sindhwani S., Zhang Y., Chen Y.Y., Macmillan P., Chan W.C.W. (2018). Quantifying the Ligand-Coated Nanoparticle Delivery to Cancer Cells in Solid Tumours. ACS Nano.

[69] Du B., Yu M., Zheng J. (2018). Transport and interactions of nanoparticles in the kidneys. Nat. Rev. Mater..

[70] Veiseh O., Gunn J.W., Zhang M. (2010). Design and fabrication of magnetic nanoparticles for targeted drug delivery and imaging. Adv. Drug Deliv. Rev..

[71] Blanco E., Shen H., Ferrari M. (2015). Principles of nanoparticle design for overcoming biological barriers to drug delivery. Nat. Biotechnol..

[72] Xu F., Yuan Y., Shan X., Liu C., Tao X., Sheng Y., Zhou H. (2009). Long-circulation of hemoglobin-loaded polymeric nanoparticles as oxygen carriers with modulated surface charges. Int. J. Pharm..

[73] Papi M., Caputo D., Palmieri V., Coppola R., Palchetti S., Bugli F., Martini C., Digiacomo L., Pozzi D., Caracciolo G. (2017). Clinically approved PEGylated nanoparticles are covered by a protein corona that boosts the uptake by cancer cells. Nanoscale.

[74] Oishi M., Tamura A., Nakamura T., Nagasaki Y. (2009). A smart nanoprobe based on fluorescence-quenching PEGylated nanogels containing gold nanopartlcles for monitoring the response to cancer therapy. Adv. Funct. Mater..

[75] Nakamura T., Tamura A., Murotani H., Oishi M., Jinji Y., Matsuishi K., Nagasaki Y. (2010). Large payloads of gold nanoparticles into the polyamine network core of stimuli-responsive PEGylated nanogels for selective and noninvasive cancer photothermal therapy. Nanoscale.

[76] Manzanares-Guevara L.A., Licea-Claverie A., Paraguay-Delgado F. (2018). Preparation of stimuli-responsive nanogels based on poly(N,N-diethylaminoethyl methacrylate) by a simple “surfactant-free” methodology. Soft Mater..

[77] Wu W., Shen J., Banerjee P., Zhou S. (2011). Water-dispersible multifunctional hybrid nanogels for combined curcumin and photothermal therapy. Biomaterials.

[78] Wang H., Di J., Sun Y., Fu J., Wei Z., Matsui H., del C. Alonso A., Zhou S. (2015). Biocompatible PEG-Chitosan@Carbon Dots Hybrid Nanogels for Two-Photon Fluorescence Imaging, Near-Infrared Light/pH Dual-Responsive Drug Carrier, and Synergistic Therapy. Adv. Funct. Mater..

[79] Park J., Wrzesinski S.H., Stern E., Look M., Criscione J., Ragheb R., Jay S.M., Demento S.L., Agawu A., Licona Limon P. (2012). Combination delivery of TGF-β inhibitor and IL-2 by nanoscale liposomal polymeric gels enhances tumour immunotherapy. Nat. Mater..

[80] Look M., Stern E., Wang Q.A., DiPlacido L.D., Kashgarian M., Craft J., Fahmy T.M. (2013). Nanogel-based delivery of mycophenolic acid ameliorates systemic lupus erythematosus in mice. J. Clin. Investig..

[81] Nguyen D.H., Lee J.S., Choi J.H., Lee Y., Son J.Y., Bae J.W., Lee K., Park K.D. (2015). Heparin nanogel-containing liposomes for intracellular RNase delivery. Macromol. Res..

[82] Yu L., Dong A., Guo R., Yang M., Deng L., Zhang J. (2018). DOX/ICG Coencapsulated Liposome-Coated Thermosensitive Nanogels for NIR-Triggered Simultaneous Drug Release and Photothermal Effect. ACS Biomater. Sci. Eng..

[83] Zhang L., Xue H., Cao Z., Keefe A., Wang J., Jiang S. (2011). Multifunctional and degradable zwitterionic nanogels for targeted delivery, enhanced MR imaging, reduction-sensitive drug release, and renal clearance. Biomaterials.

[84] Zhang L., Cao Z., Li Y., Ella-Menye J.R., Bai T., Jiang S. (2012). Softer zwitterionic nanogels for longer circulation and lower splenic accumulation. ACS Nano.

[85] Li W., Liu Q., Zhang P., Liu L. (2016). Zwitterionic nanogels crosslinked by fluorescent carbon dots for targeted drug delivery and simultaneous bioimaging. Acta Biomater..

[86] Barenholz Y. (2012). Doxil® - The first FDA-approved nano-drug: Lessons learned. J. Control. Release.

[87] Turecek P.L., Bossard M.J., Schoetens F., Ivens I.A. (2016). PEGylation of Biopharmaceuticals: A Review of Chemistry and Nonclinical Safety Information of Approved Drugs. J. Pharm. Sci..

[88] Patel M.M., Goyal B.R., Bhadada S.V., Bhatt J.S., Amin A.F. (2009). Getting into the Brain. CNS Drugs.

[89] Motlaq V.F., Knudsen K.D., Nyström B. (2018). Effect of PEGylation on the stability of thermoresponsive nanogels. J. Colloid Interface Sci..

[90] Amoozgar Z., Yeo Y. (2012). Recent advances in stealth coating of nanoparticle drug delivery systems. Wiley Interdiscip. Rev. Nanomed. Nanobiotechnol..

[91] Koshkaryev A., Sawant R., Deshpande M., Torchilin V. (2013). Immunoconjugates and long circulating systems: Origins, current state of the art and future directions. Adv. Drug Deliv. Rev..

[92] Sugiyama I., Sadzuka Y. (2013). Change in the Character of Liposomes as a Drug Carrier by Modifying Various Polyethyleneglycol-Lipids. Biol. Pharm. Bull..

[93] Gref R., Lück M., Quellec P., Marchand M., Dellacherie E., Harnisch S., Blunk T., Müller R.H. (2000). “Stealth” corona-core nanoparticles surface modified by polyethylene glycol (PEG): Influences of the corona (PEG chain length and surface density) and of the core composition on phagocytic uptake and plasma protein adsorption. Colloids Surf. B Biointerfaces.

[94] Jokerst J.V., Lobovkina T., Zare R.N., Gambhir S.S. (2011). Nanoparticle PEGylation for imaging and therapy. Nanomedicine.

[95] Suk J.S., Xu Q., Kim N., Hanes J., Ensign L.M. (2016). PEGylation as a strategy for improving nanoparticle-based drug and gene delivery. Adv. Drug Deliv. Rev..

[96] Mauri E., Cappella F., Masi M., Rossi F. (2018). PEGylation influences drug delivery from nanogels. J. Drug Deliv. Sci. Technol..

[97] Li S.D., Huang L. (2010). Stealth nanoparticles: High density but sheddable PEG is a key for tumor targeting. J. Control. Release.

[98] Yang Q., Lai S.K. (2015). Anti-PEG immunity: Emergence, characteristics, and unaddressed questions. Wiley Interdiscip. Rev. Nanomed. Nanobiotechnol..

[99] Oishi M., Hayashi H., Iijima M., Nagasaki Y. (2007). Endosomal release and intracellular delivery of anticancer drugs using pH-sensitive PEGylated nanogels. J. Mater. Chem..

[100] Oishi M., Hayashi H., Uno T., Ishii T., Iijima M., Nagasaki Y. (2007). One-Pot Synthesis of pH-Responsive PEGylated Nanogels Containing Gold Nanoparticles by Autoreduction of Chloroaurate Ions within Nanoreactors. Macromol. Chem. Phys..

[101] Oishi M., Hayashi H., Itaka K., Kataoka K., Nagasaki Y. (2007). pH-Responsive PEGylated nanogels as targetable and low invasive endosomolytic agents to induce the enhanced transfection efficiency of nonviral gene vectors. Colloid Polym. Sci..

[102] Hossain M.A., Ikeda Y., Hara T., Nagasaki Y. (2013). Novel biocompatible nanoreactor for silica/gold hybrid nanoparticles preparation. Colloids Surf. B Biointerfaces.

[103] Hossain M.A., Ikeda Y., Nagasaki Y. (2011). PEGylated polyamine nanogel as a nanoreactor of silica/Gold hybrid nanoparticle preparation. J. Photopolym. Sci. Technol..

[104] Yasui H., Takeuchi R., Nagane M., Meike S., Nakamura Y., Yamamori T., Ikenaka Y., Kon Y., Murotani H., Oishi M. (2014). Radiosensitization of tumor cells through endoplasmic reticulum stress induced by PEGylated nanogel containing gold nanoparticles. Cancer Lett..

[105] Pabla N., Dong Z. (2008). Cisplatin nephrotoxicity: Mechanisms and renoprotective strategies. Kidney Int..

[106] Boyman O., Sprent J. (2012). The role of interleukin-2 during homeostasis and activation of the immune system. Nat. Rev. Immunol..

[107] Li H., Tsokos M.G., Bickerton S., Sharabi A., Li Y., Moulton V.R., Kong P., Fahmy T.M., Tsokos G.C. (2018). Precision DNA demethylation ameliorates disease in lupus-prone mice. JCI Insight.

[108] Zhao X., Deng L., Deng H., Dong A., Wang W., Zhang J. (2018). In Situ Template Polymerization to Prepare Liposome-Coated PDMAEMA Nanogels with Controlled Size, High Stability, Low Cytotoxicity, and Responsive Drug Release for Intracellular DOX Release. Macromol. Chem. Phys..

[109] Ma J., Deng H., Zhao F., Deng L., Wang W., Dong A., Zhang J. (2018). Liposomes-Camouflaged Redox-Responsive Nanogels to Resolve the Dilemma between Extracellular Stability and Intracellular Drug Release. Macromol. Biosci..

[110] Cao Z.Q., Jiang S.Y. (2012). Super-hydrophilic zwitterionic poly(carboxybetaine) and amphiphilic non-ionic poly(ethylene glycol) for stealth nanoparticles. Nano Today.

[111] Schlenoff J.B. (2014). Zwitteration: Coating surfaces with zwitterionic functionality to reduce nonspecific adsorption. Langmuir.

[112] García K.P., Zarschler K., Barbaro L., Barreto J.A., O’Malley W., Spiccia L., Stephan H., Graham B. (2014). Zwitterionic-coated “stealth” nanoparticles for biomedical applications: Recent advances in countering biomolecular corona formation and uptake by the mononuclear phagocyte system. Small.

[113] Lynch I., Dawson K.A. (2008). Protein-nanoparticle interactions. Nano Today.

[114] Banquy X., Suarez F., Argaw A., Rabanel J.-M., Grutter P., Bouchard J.-F., Hildgen P., Giasson S. (2009). Effect of mechanical properties of hydrogel nanoparticles on macrophage cell uptake. Soft Matter.

[115] Mebius R.E., Kraal G. (2005). Structure and function of the spleen. Nat. Rev. Immunol..

[116] Merkel T.J., Jones S.W., Herlihy K.P., Kersey F.R., Shields A.R., Napier M., Luft J.C., Wu H., Zamboni W.C., Wang A.Z. (2011). Using mechanobiological mimicry of red blood cells to extend circulation times of hydrogel microparticles. Proc. Natl. Acad. Sci. USA.

[117] Liu Q., Li W., Singh A., Cheng G., Liu L. (2014). Two amino acid-based superlow fouling polymers: Poly(lysine methacrylamide) and poly(ornithine methacrylamide). Acta Biomater..

[118] Mourran A., Wu Y., Gumerov R.A., Rudov A.A., Potemkin I.I., Pich A., Möller M. (2016). When Colloidal Particles Become Polymer Coils. Langmuir.

[119] Chen L.B., Zhang F., Wang C.C. (2009). Rational synthesis of magnetic thermosensitive microcontainers as targeting drug carriers. Small.

[120] Kettel M.J., Schaefer K., Pich A., Moeller M. (2016). Functional PMMA nanogels by cross-linking with cyclodextrin methacrylate. Polymer (Guildf).

[121] Danhier F., Feron O., Préat V. (2010). To exploit the tumor microenvironment: Passive and active tumor targeting of nanocarriers for anti-cancer drug delivery. J. Control. Release.

[122] Desai N. (2012). Challenges in Development of Nanoparticle-Based Therapeutics. AAPS J..

[123] Park K. (2013). Facing the truth about nanotechnology in drug delivery. ACS Nano.

[124] Kitano S., Kageyama S., Nagata Y., Miyahara Y., Hiasa A., Naota H., Okumura S., Imai H., Shiraishi T., Masuya M. (2006). HER2-specific T-cell immune responses in patients vaccinated with truncated HER2 protein complexed with nanogels of cholesteryl pullulan. Clin. Cancer Res..

[125] Kyogoku N., Ikeda H., Tsuchikawa T., Abiko T., Fujiwara A., Maki T., Yamamura Y., Ichinokawa M., Tanaka K., Imai N. (2016). Time-dependent transition of the immunoglobulin g subclass and immunoglobulin E response in cancer patients vaccinated with cholesteryl pullulan-melanoma antigen gene-a4 nanogel. Oncol. Lett..

[126] Saito T., Wada H., Yamasaki M., Miyata H., Nishikawa H., Sato E., Kageyama S., Shiku H., Mori M., Doki Y. (2014). High expression of MAGE-A4 and MHC class I antigens in tumor cells and induction of MAGE-A4 immune responses are prognostic markers of CHP-MAGE-A4 cancer vaccine. Vaccine.

[127] Uenaka A., Wada H., Isobe M., Saika T., Tsuji K., Sato E., Sato S., Noguchi Y., Kawabata R., Yasuda T. (2007). T cell immunomonitoring and tumor responses in patients immunized with a complex of cholesterol-bearing hydrophobized pullulan (CHP) and NY-ESO-1 protein. Cancer Immun..

[128] Kageyama S., Kitano S., Hirayama M., Nagata Y., Imai H., Shiraishi T., Akiyoshi K., Scott A.M., Murphy R., Hoffman E.W. (2008). Humoral immune responses in patients vaccinated with 1-146 HER2 protein complexed with cholesteryl pullulan nanogel. Cancer Sci..

[129] Barua S., Mitragotri S. (2014). Challenges associated with penetration of nanoparticles across cell and tissue barriers: A review of current status and future prospects. Nano Today.

[130] Vlastou E., Gazouli M., Ploussi A., Platoni K., Efstathopoulos E.P. (2017). Nanoparticles: nanotoxicity aspects. J. Phys. Conf. Ser..

[131] Khanna P., Ong C., Bay B., Baeg G. (2015). Nanotoxicity: An Interplay of Oxidative Stress, Inflammation and Cell Death. Nanomaterials.

[132] Tipnis N.P., Burgess D.J. (2018). Sterilization of implantable polymer-based medical devices: A review. Int. J. Pharm..

[133] Cingolani A., Casalini T., Caimi S., Klaue A., Sponchioni M., Rossi F., Perale G. (2018). A methodologic approach for the selection of bio-resorbable polymers in the development of medical devices: The case of poly(L-lactide-co-ε-caprolactone). Polymers (Basel).

